# Supplementary material for: Mapping the evolution of fertility support policies in China: A content and instrumental analysis
Source: PLoS One. 2025 Oct 9;20(10):e0332137. doi: 10.1371/journal.pone.0332137 (PMC12510515; doi:10.1371/journal.pone.0332137)
Supplement: S1 Appendix — (ZIP) [file pone.0332137.s001.zip › S1 Appendix. 226 original policy documents/106-全国人民代表大会常务委员会关于批准2017年中央决算的决议(FBM-CLI-1-316070).doc]

全国人民代表大会常务委员会关于批准2017年中央决算的决议

**发布部门：** [全国人大常委会](https://www.pkulaw.com/law/chl?Aggs.IssueDepartment=201)

**发布日期：**2018.06.22

**实施日期：**2018.06.22

**时效性：** [现行有效](https://www.pkulaw.com/law/chl?Aggs.TimelinessDic=01)

**效力级别：** [工作文件](https://www.pkulaw.com/law/chl?Aggs.EffectivenessDic=XA0105)

**法规类别：** [预算管理](https://www.pkulaw.com/law/chl?Aggs.Category=03126) [营商环境优化](https://dobusiness.pkulaw.com/)

**专题分类：** [长江经济带发展](https://www.pkulaw.com/law/chl?Aggs.SpecialType=022)

全国人民代表大会常务委员会关于批准2017年中央决算的决议
（2018年6月22日第十三届全国人民代表大会常务委员会第三次会议通过）

　　第十三届全国人民代表大会常务委员会第三次会议听取了财政部部长刘昆受国务院委托作的《国务院关于2017年中央决算的报告》和审计署审计长胡泽君受国务院委托作的《国务院关于2017年度中央预算执行和其他财政收支的审计工作报告》。会议结合审议审计工作报告，对2017年中央决算（草案）和中央决算报告进行了审查。会议同意全国人民代表大会财政经济委员会提出的审查结果报告，决定批准2017年中央决算。

国务院关于2017年中央决算的报告
（—— 2018年6月20日在第十三届全国人民代表大会常务委员会第三次会议上　 财政部部长　刘昆）

全国人民代表大会常务委员会：
　　我受国务院委托，向全国人大常委会提出2017年中央决算报告和中央决算草案，请审查。
　　**一、**2017年中央决算情况
　　2017年，在以习近平同志为核心的党中央坚强领导下，各地区各部门牢固树立政治意识、大局意识、核心意识、看齐意识，以习近平新时代中国特色社会主义思想为指导，全面深入贯彻党的十九大精神，坚持稳中求进工作总基调，坚持新发展理念，坚持以提高发展质量和效益为中心，以推进供给侧结构性改革为主线，按照党中央、国务院决策部署，认真落实十二届全国人大五次会议各项决议要求，统筹推进稳增长、促改革、调结构、惠民生、防风险各项工作，经济运行稳中向好态势持续发展，改革开放全面深化，经济结构继续优化，质量效益明显提升，风险管控稳步推进，市场信心显著增强，社会大局保持稳定。在此基础上，财政改革发展各项工作取得新进展，中央决算情况总体较好。根据[预算法](https://www.pkulaw.com/chl/0b971dd30ea57e0ebdfb.html)第[七十九条](https://www.pkulaw.com/chl/0b971dd30ea57e0ebdfb.html#tiao_79)规定，重点报告以下情况：
　　（一）中央一般公共预算收支决算情况。
　　2017年，中央一般公共预算收入81123.36亿元，为预算的103.2%。加上从中央预算稳定调节基金以及中央政府性基金预算、中央国有资本经营预算调入1633.37亿元，收入总量为82756.73亿元。中央一般公共预算支出94908.93亿元，完成预算的99.1%。加上补充中央预算稳定调节基金3347.8亿元，支出总量为98256.73亿元。收支总量相抵，中央财政赤字15500亿元，与预算持平。与向十三届全国人大一次会议报告的执行数相比，中央一般公共预算收入增加4.33亿元，主要是在库款报解整理期，进口货物增值税和消费税、专项收入等增加。中央财政调入资金增加0.02亿元，主要是中央政府性基金预算调入一般公共预算增加。中央一般公共预算支出减少168.06亿元，主要是年终据实结算项目地方上解数额增加，相应冲减中央对地方税收返还。收入增加和支出减少合计172.41亿元，已包含在上述补充中央预算稳定调节基金的3347.8亿元中。
　　在实施大规模减税降费情况下，中央财政收入增长超出预期，是我国经济持续稳中向好、发展质量和效益提升的综合反映。从收入决算具体情况看，税收收入75697.15亿元，为预算的107.5%；非税收入5426.21亿元，为预算的66.2%。税收收入中，国内增值税28166.02亿元，为预算的104.9%，主要是工业生产者价格指数涨幅高于预期；国内消费税10225.09亿元，为预算的99.6%；进口货物增值税、消费税和关税合计18968.52亿元，为预算的119.4%，主要是进口商品量价齐增，一般贸易进口大幅高于预期；企业所得税20422.79亿元，为预算的104.4%，主要是企业经营效益好于预期；个人所得税7180.73亿元，为预算的111.2%，主要是居民收入增长较快；车辆购置税3280.67亿元，为预算的115.1%，主要是汽车销售量增长超出预期；出口货物退增值税、消费税13870.37亿元，为预算的106.7%，主要是出口增长高于预期。
　　从支出决算具体情况看，中央本级支出29857.15亿元，完成预算的100.9％；中央对地方税收返还和转移支付65051.78亿元，完成预算的99.1%。中央本级支出中，教育支出1548.39亿元，完成预算的101.9%；科学技术支出2826.96亿元，完成预算的99.5%；外交支出519.67亿元，完成预算的95.2%，主要是执行中收回以前年度结余资金，相应抵减当年支出；国防支出10226.35亿元，完成预算的100%；公共安全支出1848.94亿元，完成预算的100.6%；一般公共服务支出1271.46亿元，完成预算的100.9%；粮油物资储备支出1597.48亿元，完成预算的108.2%，主要是稻谷、小麦最低收购价利息费用补贴等据实结算项目支出增加；债务付息支出3777.69亿元，完成预算的100.8%。中央对地方税收返还和转移支付中，税收返还8022.83亿元，完成预算的87.8%，主要是根据全面推开营改增试点后调整中央与地方增值税收入划分过渡方案有关税收返还政策据实安排；一般性转移支付35145.59亿元，完成预算的100.3%，其中，均衡性转移支付增长8.3%，老少边穷地区转移支付增长19.7%；专项转移支付21883.36亿元，完成预算的101.9%，主要用于加大农业、教育、社保、医疗、就业、生态环保、住房保障等重点领域投入力度。
　　中央预备费预算500亿元，实际支出60.7亿元，用于大气污染成因与治理攻关等方面，剩余的439.3亿元全部转入中央预算稳定调节基金。
　　2017年，中央一般公共预算支出结余836.07亿元（含中央预备费结余439.3亿元），加上超收2511.73亿元（含政府性基金实际多调入一般公共预算的0.37亿元），合计3347.8亿元，全部用于补充中央预算稳定调节基金。2017年初中央预算稳定调节基金余额1422.17亿元，加上上述补充的3347.8亿元、按规定用中央政府性基金结转资金补充的68.49亿元，2017年末中央预算稳定调节基金余额为4838.46亿元，2018年调入预算2130亿元后余额为2708.46亿元。
　　2017年，中央一般公共预算使用以前年度结转资金安排的支出1090.96亿元，其中，中央本级使用337.86亿元，中央对地方转移支付使用753.1亿元。中央预算周转金规模没有发生变化，2017年末余额为354.03亿元，主要用于调剂预算年度内季节性收支差额。
　　2017年，中央财政发行国债40096亿元，其中内债39812.37亿元、外债283.63亿元，筹措资金除用于到期国债还本外，其余均由中央财政统筹安排使用。发行的内债中，储蓄国债3215.28亿元，平均发行期限为4.08年；记账式国债36597.09亿元，平均发行期限为7.79年。2017年国债还本25401.36亿元，其中内债25176.18亿元、外债225.18亿元。年末国债余额为134770.15亿元，包括内债余额133447.43亿元、外债余额1322.72亿元，控制在全国人大批准的国债余额限额141408.35亿元以内。
　　2017年，中央本级“三公”经费财政拨款支出合计43.6亿元（包括基本支出和项目支出安排的经费），比预算数减少17.87亿元，主要是中央部门贯彻落实中央八项规定精神和国务院“约法三章”有关要求，从严控制和压缩“三公”经费支出，以及受客观因素影响，部分因公出国（境）、外事接待任务未实施，公务用车支出大幅减少。其中，因公出国（境）经费16.83亿元，减少1.99亿元；公务用车购置及运行费23.17亿元，减少11.87亿元；公务接待费3.6亿元，减少4.01亿元。
　　2017年，中央基建投资支出5075.74亿元，其中，中央本级支出1133.38亿元，对地方转移支付3942.36亿元，主要用于保障性安居工程、水利等“三农”建设、交通能源等重大基础设施、“一带一路”建设和区域协调发展、节能环保与生态建设等方面。
　　（二）中央政府性基金预算收支决算情况。
　　2017年，中央政府性基金收入3824.77亿元，为预算的103.2%。加上2016年结转收入298.5亿元，收入总量为4123.27亿元。中央政府性基金支出3669.19亿元，完成预算的91.6%，主要是民航发展基金等部分基金执行中一些基建项目未完成审批，不具备实施条件。其中，中央本级支出2683.6亿元，对地方转移支付985.59亿元。中央政府性基金收支决算数均与执行数持平。
　　中央政府性基金收大于支454.08亿元。其中，结转下年继续使用385.59亿元；单项政府性基金结转超过当年收入30%的部分合计68.49亿元，按规定补充中央预算稳定调节基金。
　　（三）中央国有资本经营预算收支决算情况。
　　2017年，中央国有资本经营收入1244.27亿元，为预算的96.5%。加上2016年结转收入128.03亿元，收入总量为1372.3亿元。中央国有资本经营支出1001.71亿元，完成预算的86.3%，主要是2017年国有资本经营预算收入规模下降，同时调入一般公共预算比例提高，支出规模相应减少，其中，中央本级支出766.34亿元，对地方转移支付235.37亿元。向一般公共预算调出257亿元。结转下年支出113.59亿元。中央国有资本经营收支决算数均与执行数持平。
　　按照[预算法](https://www.pkulaw.com/chl/0b971dd30ea57e0ebdfb.html)和国务院有关规定，对2017年中央财政的部分收支事项实行权责发生制核算，包括预算已经安排当年应支未支的工资和社保资金、国库集中支付年终结余以及国务院批准的其他特殊事项等。有关具体情况已向全国人大常委会专门书面报告。对上述资金，财政部将在预算执行中加强管理，及时拨付，尽快发挥资金效益。
　　2017年中央一般公共预算、政府性基金预算、国有资本经营预算的预算数、决算数及其对比分析，详见中央决算草案。草案在报国务院审定、提请全国人大常委会审查之前，已经国务院审计部门审计，并根据审计意见作了相应修改。

　　**二、**认真落实各项财税政策，有力促进了经济发展和民生改善
　　2017年，我们坚决贯彻党中央、国务院决策部署，按照全国人大有关决议要求和批准的预算，有效实施积极的财政政策，提高政策精准性有效性，加大对供给侧结构性改革、扶贫、基本民生、生态环保等重点领域保障力度，促进了经济社会持续健康发展。
　　（一）深入推进供给侧结构性改革，提升经济发展质量。
　　加强财税政策对供给侧结构性改革的支持，促进结构优化升级与新动能加快成长。一是落实“三去一降一补”重点任务。及时拨付专项奖补资金222亿元，钢铁、煤炭去产能年度目标任务超额完成，37.7万分流职工得到妥善安置。淘汰停建缓建煤电产能6500万千瓦。优化中央国有资本经营预算支出方向，大力支持中央企业处置“僵尸企业”，解决国有企业“三供一业”等历史遗留问题。落实和完善企业兼并重组、债权转让核销等相关政策，有序推进市场化法治化债转股工作，年末规模以上工业企业资产负债率下降0.6个百分点。二是深化农业供给侧结构性改革。实施绿色生态导向的农业补贴制度改革。完善粮食等重要农产品价格形成机制和收储制度，多渠道消化政策性粮棉油库存。支持完成粮改饲面积超过1300万亩，耕地轮作休耕制度试点1200万亩。全国农业信贷担保体系初步形成，累计为4.9万多个农业项目担保贷款总额超过400亿元。在13个粮食主产省选择200个产粮大县，面向适度规模经营农户开展农业大灾保险试点。加强农田水利基础设施建设，农业综合开发建设高标准农田2500万亩，高效节水灌溉面积新增2165万亩。三是培育壮大新动能。全面落实中央财政科技计划和科研项目资金管理改革举措，运用市场化方式促进科技成果转化。继续支持智能制造、工业强基、绿色制造工程和首台（套）重大技术装备保险补偿。加快集成电路、新材料等制造业重点领域发展。推动新能源汽车产业做优做强，全年推广约80万辆。启动创业投资和天使投资有关税收政策试点，在30个城市深入开展小微企业创业创新基地城市示范，积极发挥财政资金引导作用，激发社会主体活力。规范推行政府和社会资本合作（PPP）模式，鼓励民营企业参与。
　　（二）着力实施减税降费，进一步降低实体经济成本。
　　树立放水养鱼意识，多措并举，促进企业降本增效。一是持续推进减税。落实和完善营改增试点政策，简并增值税税率结构，将增值税税率由四档减至17%、11%和6%三档，将农产品等税率从13%降至11%，同时进一步改进和优化纳税服务，帮助企业应抵尽抵、充分受益。出台扩大享受企业所得税优惠的小型微利企业范围、提高研发费用在企业所得税税前加计扣除比例等政策，发挥其在促进经济转型升级、支持小微企业发展、鼓励科技创新等方面的多重效应。全年新增减税超过3800亿元。二是加大清费力度。清理规范了一批政府性基金和行政事业性收费，加上地方自主清理的政府性收费，全年涉及减收超过1900亿元。此外，相关部门出台了清理规范经营服务性收费、推进网络提速降费、降低企业用能和物流成本等措施，全年减轻社会负担超过4400亿元。三是打造收费目录清单全国“一张网”。在有关部门网站汇总公布中央和地方政府性基金和行政事业性收费目录清单、中央和地方政府定价的收费目录清单，健全乱收费投诉处理机制，用公开透明和社会监督遏制乱收费。
　　（三）加强基本民生保障，持续增强人民群众获得感。
　　践行以人民为中心的发展思想，继续增加民生投入，完善相关支出政策和机制。一是支持发展公平优质教育。统一城乡义务教育学生“两免一补”政策，1400万进城务工农民工随迁子女实现相关教育经费可携带。全面改善8万余所义务教育薄弱学校基本办学条件。支持实施第三期学前教育行动计划和高中阶段教育普及攻坚计划。完善职业教育和培训体系，推进产教融合、校企合作。博士研究生国家助学金资助标准每生每年提高了3000元。二是深化医药卫生体制改革。全面推开公立医院综合改革，取消了实行60多年的药品加成政策。开展生育保险和职工基本医疗保险合并实施试点。城乡居民基本医疗保险财政补助标准由每人每年420元提高到450元。基本公共卫生服务项目年人均财政补助标准由45元提高到50元。三是加强就业和社会保障工作。完善就业创业政策体系，促进多渠道就业创业，城镇新增就业1351万人。机关事业单位和企业退休人员基本养老金标准提高了约5.5%，并按时足额发放。出台划转部分国有资本充实社保基金实施方案。整合设立困难群众救助补助资金，惠及全国7797万困难群众。提高优抚对象等人员抚恤和生活补助标准，惠及全国860万优抚对象。支持棚户区改造开工609万套，改造农村危房190.6万户。中央财政增加阶段性财力补助规模，确保困难地区财政运转和基本民生兜底。四是推动完善公共文化服务体系。进一步改善城乡公共文化设施，49863家公益性文化设施向社会免费开放。实施中华优秀传统文化传承发展工程，支持世界文化遗产、大遗址、全国重点文物等保护项目2310个和国家级非物质文化遗产代表性项目811个。
　　（四）聚焦深度贫困地区，支持脱贫攻坚取得决定性进展。
　　贯彻精准扶贫、精准脱贫基本方略，进一步集中资源、突出重点，大力支持脱贫攻坚。一是加大扶贫资金投入。2017年中央财政补助地方专项扶贫资金861亿元，比2016年增长30.3%。有关一般性转移支付和农业、教育等领域专项转移支付也加大了对贫困地区投入力度。同时，安排地方政府债券额度600亿元，用于改善贫困地区生产生活条件。二是支持推进分类施策。将贫困县涉农资金统筹整合使用试点扩大至全部832个贫困县，全年整合资金3286亿元，由贫困县根据脱贫需要因地制宜统筹使用资金。完善资金使用管理机制，规范资产收益扶贫，积极支持产业扶贫、教育扶贫、生态扶贫等。推进农村低保与扶贫开发有效衔接，进一步加强兜底保障。三是强化扶贫资金监管。修订完善财政专项扶贫资金管理办法和绩效评价办法，开展财政扶贫资金专项检查。全年减少农村贫困人口1289万，超额完成年度减贫任务。
　　（五）大力支持生态文明建设，持续改善生态环境质量。
　　牢固树立绿水青山就是金山银山的理念，加大生态环保治理力度。一是加强大气、水、土壤污染防治。着力打好蓝天保卫战，中央财政大气污染防治资金比2016年增长43%，支持落实京津冀及周边、长三角、珠三角地区大气污染防治重点任务。在12个城市开展北方地区冬季清洁取暖试点。推动重点区域和长江、黄河、南水北调工程上游及汉江等重点流域水污染防治。在51个畜牧大县启动实施畜禽粪污资源化利用项目。支持全面开展土壤污染状况详查，强化土壤污染管控和修复。二是稳步推进生态保护。国家重点生态功能区转移支付规模达到627亿元，全国享受重点补助的县域达819个。加大祁连山生态环境保护支持力度，推动妥善解决历史遗留问题。在6省（区）开展第二批山水林田湖草生态保护和修复工程试点。落实新一轮退耕还林还草、新一轮草原生态保护补助奖励和天然林保护全覆盖政策，支持全面停止天然林商业性采伐。三是加强生态文明制度建设。完善流域上下游横向生态保护补偿机制，深入推进新安江、东江等流域开展横向生态保护补偿试点。支持建立国家公园体制、开展排污权有偿使用和交易等试点。

　　**三、**坚持依法理财，扎实做好财政改革和管理工作
　　2017年，我们深入贯彻实施[预算法](https://www.pkulaw.com/chl/0b971dd30ea57e0ebdfb.html)，落实预算审查监督重点向支出预算和政策拓展有关要求，持续深化财税体制改革，更好发挥财政在国家治理中的基础和重要支柱作用。
　　（一）增强预算法治意识，进一步严格规范预算管理。
　　将[预算法](https://www.pkulaw.com/chl/0b971dd30ea57e0ebdfb.html)各项要求落实到预算管理工作各方面，实施全面规范、公开透明的预算制度。一是完善重点支出保障机制。对教育、科技等重点支出，根据项目需求结合财力状况统筹安排、优先保障，确保发展的合理需要。二是优化转移支付结构。中央对地方一般性转移支付规模继续扩大，占转移支付总额的比重提高至61.6%，并向财力薄弱地区和贫困地区倾斜。专项转移支付项目数量减至76项，比2016年减少18项。三是健全政府预算体系。将新增建设用地土地有偿使用费等3项政府性基金转列一般公共预算，取消排污费等以收定支、专款专用的规定，中央国有资本经营预算调入一般公共预算的比例提高到22%。四是加大预算公开力度。首次在中国政府网、财政部门户网站集中公开中央预算、中央部门预算和中央对地方转移支付预算，方便人大代表和社会公众监督。公开部门预算的中央部门增加到105个，公开内容更加细化，公开时间比2016年提前8天。
　　（二）深入推进预算绩效管理，提高资金使用效益。
　　强化绩效导向，完善绩效管理体系，推动提升财政支出效能。一是扩大绩效管理范围。绩效管理从一般公共预算逐步拓展到部分政府性基金、国有资本经营预算项目以及部分政府投资基金，从项目支出拓展到部门整体支出。二是贯穿预算管理全过程。绩效目标管理实现一般公共预算中央部门本级项目和中央对地方专项转移支付全覆盖，并初步建立绩效目标与预算资金同步批复下达机制。绩效执行监控试点拓展至所有中央部门。组织中央部门对所有项目和专项转移支付开展绩效自评。三是重点绩效评价常态化。继续组织第三方对重点民生政策和重大项目开展绩效评价，优先选择教育、文化、科技、环保等领域重点项目进行评价，被评价项目数量和涉及金额均较2016年大幅增加。同时组织对中央财政经济建设支出和国家储备财政支持政策开展绩效评估。部分评估评价结果已在政策调整和预算安排时得到应用。2017年重点绩效评价和专项转移支付绩效自评工作正在抓紧进行，93个中央部门的182个项目绩效自评结果在部门决算草案中反映，15个重点政策和项目绩效评价报告随同2017年决算报送全国人大常委会参阅。
　　（三）完善财政体制和税收制度，加快建立现代财政制度。
　　加大改革力度，加强改革督察，财税体制改革向纵深拓展，现代财政制度建设取得重要阶段性成果。一是完善财政体制。分领域推进中央与地方财政事权和支出责任划分改革，外交领域改革方案出台实施，教育、医疗卫生等领域改革稳步推进。指导省级财政部门合理划分省以下财政事权和支出责任。结合财政体制、税制改革进展情况，研究制定中央和地方收入划分改革方案以及健全地方税体系改革方案。二是深化税制改革。巩固营改增成果，废止[营业税暂行条例](https://www.pkulaw.com/chl/89e5edc529fbd88bbdfb.html)，修改[增值税暂行条例](https://www.pkulaw.com/chl/ea435225c018eaaebdfb.html)。继续深化资源税改革，水资源税改革试点扩大到10个省份。出台个人收入和财产信息系统建设总体方案。三是推进税收立法。落实税收法定原则，配合做好税收立法工作，[烟叶税法](https://www.pkulaw.com/chl/41b156c3ff1e780cbdfb.html)、[船舶吨税法](https://www.pkulaw.com/chl/8225cc2614100d90bdfb.html)出台，耕地占用税法、车辆购置税法、资源税法、关税法等立法工作深入开展。
　　（四）依法规范地方政府举债行为，防范化解政府债务风险。
　　认真落实党中央、国务院有关决策部署，进一步加强地方政府债务风险防控。一是坚决遏制隐性债务增量。及时完善监管政策，印发有关文件，严格禁止地方政府借融资平台公司、PPP、政府投资基金、政府购买服务等名义变相举债。二是严查违法违规融资担保行为。加强地方政府债务常态化监督，组织核查部分市县和金融机构违法违规融资担保问题，依法查处，问责到人，对典型案例公开通报。三是支持地方依法举债。在严堵“后门”的同时，进一步开好“前门”。强化地方政府债务限额管理，继续开展地方政府债务风险评估和预警。试点发行地方政府土地储备专项债券、政府收费公路专项债券，保障重点领域合理融资需求。2017年，地方政府发行一般债券2361935亿元，偿还当年到期一般债券2414.5亿元，年末地方政府一般债务余额103631.79亿元。地方政府发行专项债券19961.59亿元，当年没有专项债券到期，年末地方政府专项债务余额61468.01亿元。2017年末，地方政府债务余额合计165099.8亿元，控制在全国人大批准的余额限额188174.3亿元以内。

　　**四、**强化问题导向，进一步提高财政预算管理水平
　　2017年预算执行效果较好，同时还存在一些需要解决的问题，审计也指出了存在的不足。
　　我们对此高度重视，特别是对审计查出问题严肃认真进行整改，深入研究和采纳审计提出的建议，深化改革，完善机制，努力提升财政预算管理水平。一是深化部门预算管理改革。加强部门全口径预算管理，加大当年预算和结转资金的统筹力度。完善预算支出定额标准体系，健全支出标准动态调整机制。强化项目支出预算管理，严格项目立项程序，建立项目动态评估清理机制。扩大项目预算评审范围，推进提高项目预算编报质量。硬化预算约束，严控预算追加。二是加强预算统筹安排。明确一般公共预算统一保障履行公共服务职能。加强政府性基金预算、国有资本经营预算和一般公共预算的衔接，努力减少资金安排交叉重复。进一步完善和丰富国有资本经营预算编报，增强完整性，提高国有资本经营收益上缴财政的比例。推动社会保险基金精算平衡，提高相关基金自求平衡能力。三是规范转移支付管理。与财政事权和支出责任划分改革相衔接，研究完善中央对地方转移支付体系。增强一般性转移支付在缩小地区间财力差异方面的作用，提高地方统筹使用资金能力。严格专项转移支付资金分配和使用管理，健全专项定期评估和退出机制，加强对专项的绩效考核。四是全面实施预算绩效管理。制定全面实施绩效管理的指导性文件。逐步将绩效管理覆盖所有财政资金，深度融入预算编制执行。健全绩效指标和标准体系，改进绩效评价方式方法，推进绩效信息公开，加强绩效监督问责。五是完善中央基建投资管理。提高中央基建投资预算编制的准确性和到位率。推动中央基建投资计划提前下达，进一步做好投资计划和预算编制下达的衔接。健全中央基建投资绩效管理制度。六是防范化解地方政府债务风险。采取有效措施，坚决遏制隐性债务增量，积极稳妥化解隐性债务存量。加强监督问责，从严整治举债乱象，严肃查处违法违规融资担保行为。七是改进决算编报。贯彻落实[预算法](https://www.pkulaw.com/chl/0b971dd30ea57e0ebdfb.html)和中办印发的《关于人大预算审查监督重点向支出预算和政策拓展的指导意见》，进一步改进决算编报工作。推动中央一般公共预算支出按经济性质分类编列到款。
　　委员长、各位副委员长、秘书长、各位委员，今年以来，我国经济运行延续稳中向好态势，财政收入持续增长，重点支出得到较好保障。1— 5月，全国一般公共预算收入86650亿元，同比增长12.2%，其中中央一般公共预算收入41880亿元，同比增长15.3%；全国一般公共预算支出82695亿元，同比增长8.1%，其中中央本级支出11945亿元，同比增长7%。考虑到国内外环境仍存在一些不确定不稳定因素，价格因素增收作用可能减弱，实施新一轮减税降费政策等影响，后期财政收支平衡依然存在压力，实现全年预算目标需要付出较大努力。
　　下一步，我们将认真贯彻落实中央经济工作会议和十三届全国人大一次会议精神，坚持稳中求进工作总基调，坚持新发展理念，围绕推动高质量发展，实施好积极的财政政策。重点做好以下工作。一是加强预算执行管理。根据党和国家机构改革后有关中央部门设置和职能配置情况，调整优化预算管理，强化支出项目统筹，加强与有关部门的协调配合，促进事业发展和提高政府整体效能。从严管理财政资金，保障资金安全。强化预算执行动态监控和分析，督促地方和部门加快预算执行进度，提高财政资金使用效益。落实好深化增值税改革、进一步减少涉企收费等措施。二是支持打好三大攻坚战。继续依法加强限额内地方政府债务管理，着力防控地方政府隐性债务风险。严格坚持现行扶贫标准，强化扶贫资金整合，进一步提高整合深度和质量，实施扶贫资金绩效管理。研究细化相关举措，集中资金支持打好污染防治重大战役，促进生态环境质量总体改善。同时，坚定实施乡村振兴、区域协调发展等国家重大战略。三是做好民生保障工作。坚持尽力而为、量力而行，着力保基本、兜底线。出台实施企业职工基本养老保险基金中央调剂制度方案，实现基金安全可持续和财政负担可控，确保各地养老金按时足额发放。指导地方完善县级基本财力保障机制，进一步下沉财力，增强困难地区和基层政府保工资、保运转、保基本民生的能力。四是加快财税体制改革。加快出台修改后的[预算法实施条例](https://www.pkulaw.com/chl/089bf3d5ca379aecbdfb.html)。研究制定交通运输、环境保护等领域中央与地方财政事权和支出责任划分改革方案。推进综合与分类相结合的个人所得税制度改革。推进地方税体系建设。抓紧制定中央与地方收入划分改革方案。抓紧完成消费税法、印花税法、关税法等草案的起草工作。稳步推进中央党政机关和事业单位经营性国有资产集中统一监管试点。建立健全国有资产报告机制，推进落实金融企业国有资产专项报告和国有资产综合报告相关工作。
　　委员长、各位副委员长、秘书长、各位委员，我们将更加紧密地团结在以习近平同志为核心的党中央周围，以习近平新时代中国特色社会主义思想为指导，全面深入贯彻落实党的十九大和十九届二中、三中全会精神，认真落实本次会议审议意见，扎实做好财政预算工作，促进经济社会持续健康发展，为决胜全面建成小康社会、夺取新时代中国特色社会主义伟大胜利、实现中华民族伟大复兴的中国梦作出新贡献。

国务院关于2017年度中央预算执行和其他财政收支的审计工作报告
（—— 2018年6月20日在第十三届全国人民代表大会常务委员会第三次会议上　 审计署审计长　胡泽君）

全国人民代表大会常务委员会：
　　我受国务院委托，报告2017年度中央预算执行和其他财政收支的审计情况，请予审议。
　　根据党中央、国务院部署和审计法律法规，审计署对2017年度中央预算执行和其他财政收支情况进行了审计。审计中，我们坚持以习近平新时代中国特色社会主义思想为指导，全面贯彻党的十九大和十九届二中、三中全会精神，认真执行十二届全国人大五次会议决议，落实全国人大常委会审议意见和全国人大财经委审查意见，贯彻新发展理念，落实“三个区分开来”的重要要求，聚焦打好三大攻坚战，着力推动供给侧结构性改革和“三去一降一补”任务落实，深入推进党风廉政建设和反腐败斗争，促进经济社会持续健康发展。现将审计结果报告如下：
　　2017年，在以习近平同志为核心的党中央坚强领导下，各地区各部门认真贯彻党中央、国务院决策部署，全面深化改革开放，创新和完善宏观调控，高质量发展取得良好开端。从审计情况看，中央预算执行情况总体较好，财政保障能力进一步增强。
　　——经济保持平稳运行，质量效益持续提升。经济运行保持在合理区间，经济实力稳步提高。以供给侧结构性改革为主线，持续深化放管服改革，扎实推进“三去一降一补”，大力发展新兴产业，深入实施创新驱动发展战略，结构调整对经济发展的支撑作用明显。
　　——着力打好三大攻坚战，补短板强弱项取得明显成效。强化地方政府债务限额管理，加强金融监管统筹协调。中央财政补助地方专项扶贫资金比上年增长30.3%，1289万农村贫困人口实现脱贫。中央本级节能环保、社会保障和就业、教育等支出分别增长18.6%、12.4%、7%，享受国家重点生态功能区转移支付的县域增至819个。
　　——积极财政政策有效实施，财税体制改革不断深化。中央财政一般公共预算收入和支出分别增长7.1%、5.3%，保障了重点领域发展需要。营改增全面推开，小微企业税收优惠措施有效落实，全年为各类市场主体减负超1万亿元。13个省制定了中央与地方财政事权和支出责任划分改革方案。
　　——审计查出问题整改成效较好，体制机制进一步健全。对上一年度审计查出的问题，各地区、部门和单位按照党中央、国务院部署，认真落实整改主体责任，审计署按项、逐条跟踪督促。目前，审计查出的突出问题都得到了整改，推动制定完善规章制度2000多项。

　　**一、**中央决算草案和预算管理审计情况
　　重点审计了财政部具体组织中央预算执行和编制中央决算草案、发展改革委组织分配中央基建投资情况。财政部编制的中央决算草案列示的决算收支表明，2017年，中央一般公共预算收入总量82756.73亿元、支出总量98256.73亿元，赤字15500亿元；预算稳定调节基金年底余额4838.46亿元；政府性基金收入3824.77亿元、支出3669.19亿元；国有资本经营收入1244.27亿元、支出1001.71亿元。从审计情况看，财政部、发展改革委深化财税体制改革，加强预算和计划管理，优化财政支出结构，优先保障基本民生和重点项目，预算管理水平和财政资金使用绩效有所提高。存在的主要问题：
　　（一）中央决算草案编制不够准确完整。
　　1．少计中央一般公共预算收支20.13亿元。一是以退库方式安排资金16.53亿元，未作收支反映；二是国际金融组织赠款未纳入预决算，涉及收支3.6亿元；三是核算不准确，将收回的农业保险保费补助支出3.01亿元，抵减税收返还支出3.01亿元。
　　2．两个重大事项披露不充分。未单独反映政府投资基金中央财政2017年出资358.11亿元及收益分配，6964亿元中央特别国债2017年8月到期续作情况。
　　3．部分收支事项列示不细化。收入方面，未分税种列示进出口环节增值税、消费税收入及退税情况。支出方面，执行中调整为补助地方支出的70.11亿元直接列入“其他支出”；中央基本建设支出中，部分本级支出只列示到类级科目，转移支付未按投资专项分项列示。
　　审计指出问题后，财政部在决算中增加了中央对地方税收返还3.01亿元，披露了政府投资基金注资和特别国债续作情况，细化了进出口环节税收及退税、补助地方其他支出以及中央基本建设支出等情况。
　　（二）预算分配管理存在薄弱环节。
　　1．预算安排与存量资金盘活统筹衔接不够。一是财政部对累计结转42.3亿元的48个项目，仍安排预算49.01亿元，年底结转增至59.11亿元；13个部门及18家所属单位结余3.43亿元未及时上交。二是发展改革委安排支持的18个项目无法按计划实施或超过1年未开工，地方未及时申请调整，导致中央基建投资2.55亿元闲置。三是17个部门未将4160个项目结转资金86.65亿元统筹纳入部门预算。
　　2．预算分配标准不够明确或执行不严格。一是未明确规定分配因素或权重，涉及24项专项转移支付。二是分配标准不细化或不合理，涉及1项专项转移支付、3个投资专项，资金33.33亿元。三是分配中未严格执行规定的办法和标准，涉及一般性转移支付、7项专项转移支付、11个投资专项，资金2501.12亿元，如发展改革委超出标准向16个项目多安排4794.9万元，低于标准向23个项目少安排5163.4万元，向不符合条件的17个项目分配1.26亿元。
　　3．部分预算安排和下达不够规范。一是预算级次不清，在5个部门的项目支出中安排补助地方支出20.86亿元；有133.38亿元对地方的基建投资，在执行中被调整为中央本级支出。二是预算编制不细化，22项专项转移支付预算编制时未落实到具体地区，其中11项连续2年以上未落实。三是预算下达不及时，9个部门的19个项目预算至12月才追加，下达的1.46亿元当年全部结转。转移支付中，财政部和发展改革委分别有3833.03亿元、212.18亿元未在规定时间内下达，40项专项提前下达比例偏低，其中24项全部未提前下达。
　　4．国有资本经营预算范围不够完整。截至2017年底，中央部门所属事业单位的4900余户企业中，有4100余户（占83%）尚未纳入国有资本经营预算范围，当年净利润约240亿元。
　　5．部门间对接还不够顺畅。发展改革委未及时将对地方的投资专项予以细化，财政部估算编制的年初预算与发展改革委实际下达计划差异较大，有18个地区多346.77亿元，其余18个地区少213.15亿元；财政部未及时将发展改革委安排的35.68亿元投资计划批复到部门年初预算，年底形成结转15.07亿元。抽查3519.61亿元投资发现，从下达计划到下达预算平均耗时74天。
　　（三）转移支付管理仍不够完善。
　　1．具有指定用途的转移支付占比仍较高。2017年中央对地方转移支付中，专项转移支付占比下降1个百分点，但一般性转移支付中有37项12434.42亿元资金指定了用途，加上专项转移支付，地方无法统筹使用的资金占比仍达60%。
　　2．专项转移支付退出机制不完善。现有76项专项转移支付中，52项未明确规定实施期限或退出条件，支出项目只增不减的格局尚未根本改变。定期评估覆盖面较窄，2017年仅对32项专项转移支付开展评估，其中11项评估内容不完整。
　　3．部分转移支付安排交叉重叠。主要是对同类事项或支出通过多个渠道安排资金。一是多本预算安排，如对旅游基础设施建设等事项，一般公共预算和政府性基金预算分别安排24.6亿元、29.76亿元。二是多个部门安排，如对11类具体事项，财政部、发展改革委分别安排1570.6亿元、472.6亿元。三是多个专项安排，财政部2项专项转移支付和发展改革委2个投资专项，均安排资金用于高标准农田建设；发展改革委2个投资专项安排同一地区重大项目前期工作经费，涉及1950万元。
　　（四）部分财税领域改革有待深化。
　　1．预算绩效评价不到位。主要是实际推进中存在评价覆盖面小、指标不细化、自评不严格等问题。2017年，财政部对16个中央本级项目支出进行了绩效评价，项目资金覆盖面仅1.38%，结果也未全部公开；发展改革委未按要求对分配的中央本级项目开展绩效目标管理。抽查还发现，8个部门24个项目的绩效目标脱离实际或指标不够细化；8个部门14个项目自评结果不实，其中资金使用率自评100%的2个项目实际支出为零。
　　2．中央政府投资基金管理不规范。由于基金设立采取一事一议方式，对政策目标、职能划分等缺乏统筹规划，审计发现国家新兴产业创业投资引导基金和先进制造产业投资基金分别投资同一公司4亿元、15亿元。个别基金未充分发挥政策引导作用，如中国政企合作投资基金2016年3月成立，截至2017年底到位资金中有639亿元（占88.7%）未投资项目，用于购买理财产品。
　　3．税收优惠政策后续管理不到位。财政部尚未建立税收优惠政策定期评估机制，无法全面掌握执行效果，不利于政策调整完善。如固定资产加速折旧政策未覆盖一些新能源、新材料等新兴制造业，2017年由国税征管的制造业企业仅有1.71万户享受了此项优惠。
　　审计指出问题后，财政部出台了税收优惠政策定期评估相关规定。对其他问题，财政部、发展改革委等部门正在整改。

　　**二、**中央部门预算执行审计情况
　　重点审计了57个中央部门本级及所属365家单位，涉及财政拨款预算2115.99亿元（占这些部门财政拨款预算总额的35.28%）。审计结果表明，2017年度部门预算管理不断加强，预算执行总体较好，财政资金使用绩效有所提高。审计发现的主要问题：
　　一是预决算编报还不够准确。涉及35个部门和81家所属单位、金额35.04亿元。其中：6个部门和9家所属单位编报预算时，虚报项目内容或人员等多申领资金2.16亿元；11个部门和11家所属单位存在代编预算等问题12.62亿元；28个部门和64家所属单位的决算草案存在多列支出、少计收入等问题，涉及20.26亿元。
　　二是资金资产管理还不够规范。涉及23个部门和89家所属单位、金额11.25亿元。其中：1个部门和10家所属单位账外存放或通过虚假票据等套取资金4409.55万元；4个部门和11家所属单位违规理财或出借资金等2.58亿元；7个部门和31家所属单位未严格执行“收支两条线”规定，涉及租金等收入5.4亿元；16个部门和40家所属单位部分资产违规处置或闲置浪费等，涉及2.83亿元。
　　三是“三公”经费和会议费管理不严问题在一些部门依然存在。涉及34个部门和101家所属单位、金额5721.71万元。因公出国（境）方面，5个部门和1家所属单位超计划、超限量、超人数安排出国团组113个；4个部门和10家所属单位无预算、超预算列支或转由其他单位承担出国（境）费用852.86万元。公务用车方面，6家所属单位未按规定完成公车改革；10个部门和25家所属单位超标准超编制、违规或变相配备、未按规定使用公务用车等240辆；2个部门和2家所属单位无预算、超预算、超标准支出公务用车购置及运行维护费649.76万元。会议和培训管理方面，11个部门和18家所属单位无计划、计划外召开会议或举办培训等578个；3个部门和17家所属单位违规在非定点饭店或京外召开会议98个；12个部门和12家所属单位超预算、超标准、超范围列支，或转由其他单位承担会议费、培训费2808.91万元。此外，还有10个部门和29家所属单位存在违规发放津贴补贴、违规兼职或取酬等问题，涉及1204.04万元。
　　四是一些部门和单位依托管理职能或利用行业影响力违规收费。涉及5个部门和75家所属单位、金额1.69亿元。其中：62家所属单位通过组织资格考试、开展检测等取得收入1.61亿元；5个部门和14家所属单位开展评比表彰、举办论坛等收费829.56万元。
　　审计指出问题后，有关部门和单位通过归还资金原渠道、上缴国库、调整账目等方式整改5.06亿元，清退超编制或违规占用其他单位公务用车10辆，出台和完善制度5项。

　　**三、**重大专项资金和民生工程审计情况
　　（一）乡村振兴相关资金审计情况。重点调查了9个省农村土地承包经营权确权、28个省农业信贷担保体系建设和8个省畜禽养殖业粪污资源化利用等3项重点工作的推进情况。截至2017年底，全国已完成土地确权面积11.59亿亩，投入584亿多元设立农业信贷担保机构528个，畜禽粪污处理综合利用率有所提高，但有些工作进展较慢，部分政策落实与要求还有较大差距。截至2018年2月底，9个省土地确权资金累计结存61.46亿元（占收到额的44%），部分闲置1年以上，7个省土地承包经营权证颁证率最高12%、最低为零；抽查7个省级农业信贷担保机构2017年5月后开展的66亿多元担保业务中，符合规定标准的仅占22%、最低7%；7个省未出台粪污资源化利用绩效考核办法，由于养殖业与种植业对接困难，抽查5个县2017年产生的1395.61万吨畜禽粪污中，仅34.2万吨（占2.5%）作为有机肥利用。审计指出问题后，有关地方和部门正在整改。
　　（二）医疗保险基金审计情况。截至2017年底，审计的9个省基本建立大病保险、疾病应急救助等制度，将4100多家医院纳入跨省异地就医结算系统，城乡居民政策范围内住院费报销比例比上年平均提高2个百分点，但一些地方统筹和保障还不到位，3个省尚未实现新农合与城镇居民医保药品目录、筹资政策等“六统一”；9个省43个市县的125.9万人未参加城乡居民医保或未能按政策享受新农合保费补贴。4个省21家企业的166.65万名职工医保基金未实行属地管理；2个省的3个市未及时拨付医保费用9500多万元。审计指出问题后，相关地方已拨付大病保险1319万元。
　　（三）工程项目薪酬发放审计调查情况。审计抽查了19个省188个工程项目，推动为3.4万余名农民工追回欠薪5.94亿元、问责182人。从抽查情况看，欠薪的原因主要是有的地方超财力投资工程、前期工作不到位、工程款拨付不及时；一些施工企业为竞揽工程，超越自身实力先行垫付建设资金，后因资金不足而欠薪；落实工资保证金、工资专用账户等保障政策也不到位。
　　（四）结构调整专项奖补资金审计情况。重点抽查了22个省和2户企业收到的工业企业结构调整专项奖补资金管理使用情况。总的看，相关资金管理使用逐步规范，有力推动了化解过剩产能，但仍发现有4家地方单位和1户企业将7亿元违规用于偿债、理财、发放在职人员工资等，2户企业以虚假材料申报违规获取2954万元；有17个省多报或少报去产能职工安置人数5.07万人，加之有些地方未按要求明确奖补范围，导致16.91亿元结存或无法分配。审计指出问题后，有关地方和企业正在整改。
　　（五）全国保障性安居工程跟踪审计情况。重点抽查了全国1.77万个项目，涉及投资2.52万亿元。从审计情况看，各地积极探索建立和完善多主体供给、多渠道保障、租购并举的住房制度，大力推进项目建设，群众居住条件明显改善，但部分资金和住房仍存在被挤占挪用、违规获取或空置等问题，涉及资金30.84亿元、住房16.87万套，还有620.46亿元未及时安排使用。后续监管也未及时跟进，3.53万户不再符合保障条件的家庭仍享受住房2.75万套和补贴1384.43万元；1211个项目建成后因手续不完备无法办理竣工验收备案。审计指出问题后，有关地方已追回或盘活资金等26.07亿元，取消或调整保障待遇1.68万户，收回和加快分配住房8602套。

　　**四、**三大攻坚战相关审计情况
　　为贯彻落实党中央、国务院部署，审计署先后印发了关于加强审计监督推动打好三大攻坚战的3个实施意见，各项审计都高度关注相关重点任务推进情况。
　　（一）防范化解重大风险相关审计情况。重点关注了地方政府债务、金融运行和基本养老保险基金支付等风险及防范情况。
　　1．地方政府债务风险防控情况。在2017年持续组织开展地方政府债务审计基础上，2018年一季度重点审计了5个省本级、36个市本级和25个县级政府债务的管理情况，截至2018年3月底这些地方出台债务管理制度132项。2017年7月全国金融工作会议后，相关地区风险防范意识进一步增强，举债冲动得到有效遏制，违规举债问题明显减少，目前债务风险正得到有序有效防控，但仍发现5个地区2017年8月以后违规举债32.38亿元，还有3个地区政府违规提供担保9.78亿元；11个地区发行的政府债券筹集资金中，有39.22亿元结存在财政部门，其中29.2亿元闲置1年以上。一些地方落实债务管理要求还不到位，有14个地区化解债务方式过于简单，造成重点项目建设资金接续不畅等；5个省本级尚未筹建债务大数据监测平台，14个市县未按要求全面摸排融资担保行为或未按时整改到位，5个市县未将政府购买服务等事项纳入监测范围。审计指出问题后，有关地方正在整改。
　　2．金融风险防控情况。各项审计都关注了金融风险防范化解情况。从审计情况看，金融机构和有关企业风险防控工作持续加强，监管部门也加大了对高风险行业和领域整治力度。审计发现，9家大型国有银行违规向房地产行业提供融资360.87亿元，抽查的个人消费贷款中也有部分实际流入楼市股市；一些金融机构通过发行短期封闭式理财产品吸收资金，用于投资长期项目等，存在期限错配风险。网络贷款领域风险管控还不到位，抽查在重庆注册的29家网络小额贷款公司中，有12家主要在浙江等地经营，以规避实际经营地区的准入审查，由此导致的网络客户信用识别不精准、非法冒用他人名义借贷等问题较突出；抽查60家“现金贷”机构发现，其中40家无从业资质，有的以学生为主要客户，存在较大隐患。审计指出问题后，有关方面正在整改，8家银行已收回贷款等45.86亿元。
　　3．基本养老保险基金支付风险防控情况。截至2017年底，审计的9个省企业职工基本养老保险基金累计结余1.82万亿元，同比增长12.8%，总体收大于支，但省级统筹在一些地方落实还不到位，6个省有60个市2017年当期结余554.25亿元，而省内其他45个市当期缺口460.55亿元。32个市存在通过占用财政资金和失业保险基金、向银行贷款等方式弥补基金缺口的现象。还有4个省存在违规调整缴费基数下限等问题，造成少缴或欠缴保费11.94亿元。审计指出问题后，相关地方和单位已收回财政资金8000多万元。
　　（二）扶贫政策落实和资金管理审计情况。组织重点审计了21个省的312个国定贫困县（含114个深度贫困县），抽查2500多个乡镇、8000多个行政村，走访2.9万多户家庭，涉及资金1500多亿元。总的看，各地区各部门积极贯彻落实党中央、国务院决策部署，脱贫攻坚取得重大进展，贫困地区生产生活条件显著改善，贫困群众获得感明显增强。审计发现的主要问题：
　　1．一些地方扶贫工作还不够扎实。主要表现为形式主义、弄虚作假及违反中央八项规定精神。其中：37个县把10.92亿元投向企业、合作社和大户，但未与贫困户建立利益联结；13个县将3.21亿元产业扶贫等“造血”资金直接发放给贫困户；5个县将540多万元用于景观修建、外墙粉饰等；4个县在易地扶贫搬迁范围、建设标准等环节层层加码，形成资金缺口2.97亿元，原定任务也未完成；12个市县存在虚报脱贫数据等问题；12个市县扶贫工作中存在超标准接待问题，涉及1700多万元。
　　2．有的扶贫政策落实不够精准。96个地区建档立卡数据不够完整、准确，50多万贫困户（人）未按规定享受助学金、危房改造等补贴2.86亿元；23个地区易地扶贫搬迁规划不合理、配套不齐全等，影响2万多贫困户（人）安置；30个地区脱贫标准把握不准或未按规范验收，将“两不愁、三保障”未落实的8万多贫困人口认定为脱贫。“三区”教师扶贫专项计划落实不到位，2017年有160多个贫困县未获安排支教教师，3万多名支教教师中有71%是从贫困县选派到贫困县。
　　3．一些地方涉农资金统筹整合试点推进不畅。25个县的19亿多元未按规定纳入统筹整合范围；23个县纳入统筹整合的70多亿元实际仍专款专用；4个县将已挪作他用的1.8亿元纳入统筹整合范围，影响数据真实性。
　　此外，部分扶贫资金和项目监管仍较粗放，有28.11亿元被骗取套取或挪用，举借的11.75亿元闲置，还有261个项目（投资2.88亿元）长期闲置或未达目标。
　　审计指出问题后，有关部门和地方已追回或盘活6.64亿元，完善规章制度74项，处理处分231人。
　　（三）污染防治资金和项目审计情况。重点开展了长江经济带生态环境保护审计和46个地区领导干部自然资源资产离任审计。审计结果表明，有关地区和领导干部认真落实自然资源资产管理和生态环境保护责任，长江经济带“共抓大保护、不搞大开发”取得一定成效，生态环境质量有所改善，但仍发现一些问题：
　　1．生态环境保护协同机制仍不够健全，跨地区、跨领域防控仍是难点。如湖北重要水源地王英水库地跨3市县，上游地区污染治理不够到位，下游虽多次通报上游，始终未有效解决。网络销售电鱼机等问题缺乏监管，影响对非法电鱼的打击整治效果，近4年非法电鱼案件年均增长8.8%，破坏长江流域水生态系统健康。
　　2．开发管控不够到位，生态修复尚未达到预期。抽查发现，长江经济带11个省现有开发区中，8个是2016年以来未经批准新建的，62个坐落在国家重点生态功能区或与自然保护区等重叠，176个未建设污水集中处理设施或配套设施不完备；小水电开发强度较大，10个省累计建成2.41万座，平均每万平方公里118座，最小间距仅100米。持续整治的5个国家重要湖泊，由于统筹治理不到位等，2017年湖水水质仍为Ⅳ类及以下。
　　3．部分资金和项目绩效不高。有关地区少征或违规使用相关资金177.25亿元，62.79亿元专项资金闲置1年以上；206个污染防治和生态修复项目未按期开（完）工，43个项目建成后效果不佳。
　　审计指出问题后，有关地方已整改14.14亿元，完善制度62项，处理处分105人。

　　**五、**重大政策措施落实跟踪审计情况
　　2017年以来，全国共跟踪审计7.53万个项目，抽查7.66万个单位，推动下达和落实、收回沉淀和统筹安排资金506.59亿元，促进减少或清退收费等122.08亿元，推动新开（完）工或加快进度项目5789个，促进建立健全规章制度1076项，相关地方和部门处理处分2514人。近期审计发现的主要问题：
　　（一）制造业增值税抵扣链条还需进一步完善。调查营改增等落实情况发现，实施营改增试点以来，累计减税超过2万亿元，其中简易征收等过渡性优惠政策对于保证试点行业税负只减不增发挥了积极作用，但也增加了开具增值税普通发票的上游企业数量，使一些接受增值税普通发票的下游制造企业无法用于抵扣，相关政策还需进一步完善。
　　（二）一些地方营商环境不够优化。11个部门和地区的11项放管服事项改革不到位，2个地区的3家单位违规继续征收行政事业性收费1387.74万元，12个省的27个部门向2000多家单位收取或转嫁审批前置中介等费用4.57亿元，还有9个地区的23家单位违规预留或未及时清退企业保证金16.72亿元。
　　（三）一些重大工程项目推进较慢。对“十三五”规划纲要中的20个重大工程项目（占规划总数的12%）审计发现，截至2018年3月底，由于未及时制定具体方案或任务清单、未分解落实责任、未出台配套政策等，10个项目的41项任务有部分具体事项进展较慢，不利于任务如期完成。
　　（四）创新创业相关制度还不完善。主要是科研项目预算和经费管理不完全适应科研创新的需要，科研考核评价机制难以有效发挥激励作用，重论文和外语、轻创新成果实际应用和转化的问题仍然存在。抽查4个高校双创示范基地2016年4614项专利授权发现，平均转化率不足7%。抽查11个省36支创业投资引导基金中，有6支从未对外投资，涉及资金7.8亿元。
　　（五）政务信息系统整合和安全管理要求未有效落实。2017年7月至9月，对71家中央部门3188个政务信息系统（总投资375.19亿元）审计发现，4个部门信息系统存在重复开发问题，416个系统仅在司局甚至处室内部应用，部分系统未确定安全等级或未按规定进行安全管理。
　　（六）淘汰落后产能不够到位。吉林1处30万吨/年煤矿产能未按规定退出；云南违规新增水泥产能450万吨。
　　审计指出问题后，有关地方和部门正在整改。

　　**六、**金融和企业审计情况
　　主要审计了9家大型国有银行和35户央企。结果表明，这些金融机构和企业能够贯彻落实相关政策措施，不断深化改革，完善法人治理结构，提升经营管理水平，实现国有资产保值增值。审计发现的主要问题：
　　（一）部分改革试点任务未及时完成。4户纳入国有资本投资运营公司试点、落实董事会职权试点或混合所有制改革试点的企业未及时制定有关工作方案或具体细则；4户未在规定时间内完成本企业全面深化改革实施方案的制定完善工作。
　　（二）违规决策和经营问题仍较突出。有3家大型国有银行违反程序、违规承诺等办理信贷、理财业务945.99亿元，违规向“两高一剩”行业等提供融资1222.29亿元，虚增存款规模45.1亿元。审计35户央企发现，2016年多计利润28.65亿元，占同期利润的0.77%；有175项重大经济决策事项涉嫌违规或盲目决策等，556项经营管理事项不够规范，共造成资产损失、损失风险和闲置等203.67亿元。
　　（三）违反中央八项规定精神等问题依然存在。30户央企和3家大型国有银行购买礼品、旅游和违规兼职取酬等3.06亿元；21户央企超标准购车3405.14万元、超标准乘坐交通工具768.32万元；7户央企下属单位的142人违反廉洁从业规定，存在经商办企业等问题，涉及金额6.4亿元。
　　审计指出问题后，有关金融机构和企业补缴税款9100万元，推动增收节支和挽回损失25亿元，制定和修订制度1742项，处理处分1883人。

　　**七、**审计移送的重大违纪违法问题线索情况
　　上述各项审计中，发现并移送重大问题线索300多起，涉及公职人员500多人，造成损失浪费340多亿元。主要特点：
　　（一）“微腐败”侵害群众利益。共发现此类重大问题线索50多起，主要是侵占扶贫、涉农、社保、环保等民生资金，涉案人员多为基层公职人员。如扶贫审计发现，32个市县及有关单位的153名干部（其中乡镇村干部110人）利用职务便利优亲厚友等，侵吞扶贫资金等3700多万元。
　　（二）涉众类金融乱象亟待整治。共发现非法集资、地下钱庄等重大问题线索40多起，主要是一些不法团伙通过快速增值、保本高息等虚假承诺宣传，或幕后操纵标的物市场行情，诱骗社会公众参与，如善林（上海）金融信息服务有限公司及其关联企业非法高息集资数百亿元，审计移送后，公安机关已介入侦查。
　　（三）涉税违法问题不容忽视。共发现此类重大问题线索50多起，涉嫌偷逃税款270多亿元，集中在成品油、进口农产品、白酒、手机等领域，主要是利用税收征管漏洞，通过虚构销售业务、串通定价等，虚开发票抵扣以偷逃税款。特别是在成品油领域，相关涉案企业跨省联手操作，形成一套完整的虚假抵扣链条，相关监管核查也存在薄弱环节。
　　（四）国有企业管理中滥用职权违规操作等问题仍然存在。共发现此类问题27起，造成国有资产损失等60多亿元。主要是低价转让国有资产、违规开展融资性贸易或对外出借资金等，有的企业领导人员还利用职务之便为特定关系人谋取不当利益。

　　**八、**审计建议
　　从审计情况看，对照建设现代化经济体系、实现高质量发展的要求，财政资金提质增效尚有较大空间，一些基础性关键领域改革还需加快推进，重大任务相关配套措施还需健全完善，正向激励、容错纠错和约束管控等机制还需同步发力。为此，应进一步加大改革开放力度，完善各领域政策措施和制度规则，在改革举措的落地见效上下更大功夫。
　　（一）切实提高财政资源配置效率。一是统筹考虑中央与地方财政事权和支出责任划分、税收制度改革和税收政策调整，健全地方税体系，优化转移支付制度，健全专项转移支付定期评估和退出机制，加快转移支付立法，尽快修订[预算法实施条例](https://www.pkulaw.com/chl/089bf3d5ca379aecbdfb.html)。二是进一步优化财政支出结构，加大对扶贫、污染防治、创新创业、民生等重点领域的支持力度。三是全面实施绩效管理，加大财政资金统筹力度，建立绩效评价结果与预算调整和政策调整挂钩机制，明确部门和地区主要负责人绩效评价考核第一责任人责任，切实推进财政提质增效。
　　（二）夯实基础推动高质量发展。一是巩固扩大放管服改革成效，切实遏制违规收费，完善营改增相关配套措施，加快推行营商环境评价体系，充分激发各类市场主体活力。二是建立健全各类国有资产监管法律法规体系，加快国有经济布局优化、结构调整、战略性重组，聚焦实体经济，突出和做强主业。三是深化科技体制改革，推动产、学、研融合，加大对领军企业牵头实施的重大科技项目的扶持力度，改进科研考核评价激励政策，提高科研成果转化效率。
　　（三）完善打好三大攻坚战相关措施。一是切实防范地方政府债务风险，积极稳妥化解存量、严控增量。结合金融监管机构改革，统筹抓好金融领域风险防范，完善金融机构绩效评价体系，完善对问题金融机构、非法金融活动有序处置的相关法律法规，推进网络贷款领域风险专项整治。尽快建立企业职工基本养老保险基金中央调剂制度及配套措施，积极推进养老保险全国统筹。二是深入开展扶贫领域专项整顿，严肃惩处贪污侵占、虚报冒领等行为，加强跨部门涉贫信息整合共享，精准施策、强化监管，提高扶贫资金和项目绩效。三是健全生态环境保护协同机制，进一步扩大横向生态补偿试点范围，强化生态环境修复源头治理。
　　（四）健全激励干事创业的相关配套制度。认真落实中共中央办公厅《关于进一步激励广大干部新时代新担当新作为的意见》要求，结合深化党和国家机构改革，一是全面清理、废止、修订不利于改革发展和干事创业的法规制度，对经过实践检验效果良好但尚未明确法律依据的事项，优先制定相关制度予以规范和扶持，尽快补齐制度短板。二是将完善容错纠错机制与整治不作为问题相结合，明确界定容错范围、严格落实纠错措施，进一步将“三个区分开来”的重要要求落到实处。三是加快制定和完善权责清单，依法制权限权，切实加大追责问责力度。
　　本报告反映的是中央预算执行和其他财政收支审计发现的主要问题，有关具体情况以附件形式印送各位委员，并向社会公告。下一步，审计署将认真督促整改，国务院将在年底前向全国人大常委会专题报告整改情况。
　　委员长、各位副委员长、秘书长，各位委员，我们将更加紧密地团结在以习近平同志为核心的党中央周围，高举中国特色社会主义伟大旗帜，以习近平新时代中国特色社会主义思想为指导，全面贯彻落实党的十九大和十九届二中、三中全会精神，自觉接受全国人大的指导和监督，依法履行审计监督职责，锐意进取、尽责担当，为决胜全面建成小康社会、加快推进社会主义现代化、实现中华民族伟大复兴的中国梦而奋斗！

© 北大法宝：（[www.pkulaw.com](https://www.pkulaw.com/)) 专业提供法律信息、法学知识和法律软件领域各类解决方案。北大法宝为您提供丰富的参考资料，正式引用法规条文时请与标准文本核对。
欢迎查看所有[产品和服务](https://www.pkulaw.com/helps/2.html)。法宝快讯：[如何快速找到您需要的检索结果？](https://www.pkulaw.com/helps/70.html)[法宝](https://www.pkulaw.com/helps/69.html)V6有何新特色？

扫描二维码阅读原文

**原文链接：**<https://www.pkulaw.com/chl/764ad9546abccd85bdfb.html>
